# Microwave extraction and molecular imprinted polymer isolation of bergenin applied to the dendrochronological chemical study of *Peltophorum dubium*

**DOI:** 10.1186/s13065-024-01112-7

**Published:** 2024-01-13

**Authors:** Oscar Caetano Silva-Neto, Caio Silva Assis Felix, Leonardo de Oliveira Aguiar, Mauricio Brandão dos Santos, Silvio Cunha, Jorge Mauricio David

**Affiliations:** Instituto de Química, Universidade Federal da Bahia Campus Ondina, Salvador, BA 40170280 Brazil

**Keywords:** Bergenin, Molecularly imprinted polymer, Microwave extractions, Dendrochronology, *Peltophorum dubium*, Two-level experimental design

## Abstract

This study describes methodologies for extracting and isolating bergenin, a *C*-glucoside of 4-*O*-methylgallic acid found in some plants and it presents various in vitro and in vivo biological activities. Bergenin was previously obtained from the *Pelthophorum dubim* (Fabaceae) roots with a good yield. Conventional chromatographic procedures of the CHCl_3_ soluble fraction of the MeOH extract gave 3.62% of this glucoside. An HPLC/DAD method was also developed and validated for bergenin and its precursor, gallic acid quantifications. Microwave extractions with different solvents were tested to optimize the extraction of bergenin, varying the temperature and time. MAE (Microwave Assisted Extraction) was more efficient than conventional extraction procedures, giving a higher yield of bergenin per root mass (0.45% vs. 0.0839%). Molecularly imprinted polymer (MIP) and non-imprinted polymer (NIP) based on bergenin as the template molecule, methacrylic acid, and ethylene glycol dimethacrylate were synthesized and characterized by FTIR and SEM (Scanning Electron Microscopy). Bergenin adsorption experiments using MIP and NIP followed by molecular imprinted solid phase extraction (MISPE) showed that MIP had a higher selectivity for bergenin than NIP. A dendrochronological study using the proposed method for detection and quantification of gallic acid and bergenin in five *P. dubium* growth rings of a 31-year-old heartwood and in the phelloderm and barks indicated that bergenin was more abundant in the 11–14th growth rings of the heartwood and decreased from the heartwood to the barks.

**Supplementary Information:**

The online version contains supplementary material available at 10.1186/s13065-024-01112-7.

## Introduction

**Fig. 1:**
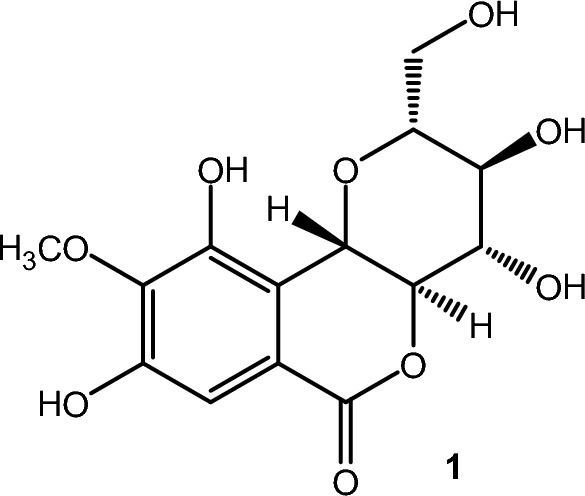
Structure of the bergenin, a *C*-glucoside derivative of gallic acid

Bergenin (**1**) is a *C*-glucoside derived from 4-*O*-methylgallic acid (Fig. 1) that occurs in several plants, and it has various biological activities, such as anti-inflammatory [1], in vivo antinociceptive [2, 3], cholinesterase inhibition [4], and serum urate reduction in hyperuricemia [5], among others. A recent review summarizes all the pharmacological and biological activities already described for this compound [6]. Thus, this compound is remarkable due to the above described in vitro and in vivo activities. Recently, the in vivo employment in rats of this compound in pretreatment, it dose-dependently relieved amnesia induced by scopolamine. It also could significantly ameliorate streptozotocin (STZ) induced behavioral deficits, inhibit the acetyl and butyril cholinesterase activities in parallel with an increase in the reduced glutatione levels in a dose-dependent way, indicating preventive and ameliorative potential of bergenin in the management of Alzheimer’s disease [7].

However, for further biological tests is necessary obtaining it in good yields. Its synthesis is possible, but the yield is lower than the isolation from natural sources [8]. Nevertheless, this compound is distributed in various species of different plant families and is usually isolated in small amounts, except few examples such as barks of Amazonian yellow uxi (*Endopleura uchi* Humiriaceae), *Cenostigma macrophyllum* (Fabaceae), and *Saxifraga atrata* (Saxifragaceae) [3, 9, 10]. In a previous screening study of crude extracts, the antimicrobial activities of different plant species from Argentina were reported, and the methanolic extract of *Peltophorum dubium* Spreng. Taub. (Fabaceae) showed inhibitory activity against *Staphylococcus aureus* [11]. The extracts of different parts of this plant were purified, and bergenin was isolated in high yields, especially from the roots and barks of *P. dubium* [12, 13]. This plant is a large tree from the Fabaceae family, commonly known as “canafístula” or “angico-vermelho”, which grows in some South American countries, particularly in Brazil’s central and southern regions. This tree is easily adaptable to tropical habitats and has economic and ornamental value. Its wood is used in civil construction, furniture, and naval industries [14]. Besides bergenin and derivatives, species of this genus is known to biosynthesize flavonoids, phenoxychromenes, terrestribisamide, and lignans [13, 15, 16].

Microwave Assisted Extraction (MAE) is a technique that is widely used because it is simple, cost-effective, allows fast extractions, and reduces solvent consumption, making the process more environmentally friendly. MAE and ultrasound assisted extraction (UAE) have increased significant focus and consideration because the costs of the instruments, especially in laboratory scale. As consequence, in the last years, there are an increment of using MAE applied to different phenolic compounds from plants and foods [17]. Molecular Imprinted Polymers (MIP) are another helpful tool that has been employed in various fields, such as membrane separations [18], dye removal in aqueous media [19], chromatographic separation of essential natural compounds like camptothecin [20], and extraction of metabolites in biological liquids [21].

As part of our ongoing investigations on the bioactivities of natural product derivatives, we present new methods for extracting and isolating bergenin (**1**) from the roots and barks of *P. dubium* with high yields. We used MIP to separate bergenin from the extracts, and we applied a two-level experimental design to optimize the MAE of bergenin. We also performed a dendrological analysis of *P. dubium* heartwood by HPLC/DAD to investigate the correlation between the presence of bergenin and other phenolic compounds and the growth of this tree.

## Results and discussion

The extract of the roots yielded 2.32% (28.63 g) from 1.23 kg of dried material employing MeOH at room temperature. The CHCl_3_ soluble fraction of this extract (8.23 g) after conventional chromatography techniques and recrystallization furnished 1.04 g of pure bergenin (3.62% of the composition of the MeOH extract and 8.39 × 10^−2^% of the dried roots. This compound was identified by UV and NMR analysis, including Heteronuclear Single Quantum Coherence Spectroscopy (HSQC) and Heteronuclear Multiple Bond Correlation (HMBC) included in Additional file (supplementary information) 1. The data obtained was also compared to the literature [3].

The procedures for extracting and purifying this compound by chromatographic methods are time expending, with various laboratory steps, and expensive. Thus, a MAE method for extraction and employment of MIP for diminishing the steps for purification was proposed. These procedures were accomplished by HPLC analysis, and this methodology was validated to bergenin and its precursor, gallic acid. The parameters (Table 1) show that the values are within the recommended by the literature. The limit of detection and quantification (LoDs and LoQs) suggest that the method has good sensitivity for the detecting these two phenolic compounds (Table 2). The HPLC flow rate and temperature parameters for robustness studies were varied in a 10% of deviation to observe whether the method resists minor and deliberate variations in the analytical parameters. The robustness was evaluated by checking the concentrations determined by the standards. No changing in the peak was observed demonstrating that the method was robust for determining these compounds.

**Table 1:** Overview of the methodology validation parameters of the gallic acid and bergenin

Parameter	Gallic acid	Bergenin
Linearity (R^2^)	0.9998	0.9996
Precision (%)	4.32	3.50
Accuracy (%)	98.83%–101.63	89.88%–102.10
LoD	0.12 µg/mL	0.01 µg/mL
LoQ	0.38 µg/mL	0.05 µg/mL
Calibration curves	y = 26114x − 18,484	y = 8400.9x − 35,785

Three different solvent systems (MeOH, EtOH, and EtOH:H_2_O) were evaluated in the optimization of bergenin extraction from the roots of *P.* dubium by MAE experiments in order to accelerate the extraction of compounds. Other two variable factors employed in the experimental design were temperature and time of extraction. The experimental domain with de-codified and absolute values of the two factors of the two level-design response was measured by the HPLC peak area and, consequently the concentration of bergenin (Tables 2 and 3).

**Table 2:** Experimental matrices for designs based on variables study in two levels employed for evaluation of the bergenin MAE using MeOH

Experiment	T (°C)	t (min)	Root mass (mg)	Extract (mg)	% Bergenin yield^a^
1	−	−	20.2	1.8	3.94
2	−	+	20.4	1.9	4.24
3	+	−	20.3	1.9	4.71
4	+	+	20.1	1.2	7.11
5	0	0	20.5	2.8	3.07
6	0	0	20.3	2.4	3.82
7	0	0	20.1	2.7	3.24

^a^In relation to the extract

**Table 3:** Experimental matrices for designs based on variables study in two levels employed for evaluation of the bergenin MAE employing EtOH:H_2_O (6:4)

Experiment	T (°C)	t (min)	Root mass (mg)	Extract (mg)	% Bergenin yield^a^
1	−	−	20.3	1.7	2.22
2	−	+	20.5	1.8	1.96
3	+	−	20.2	1.8	2.93
4	+	+	20.4	1.1	5.13
5	0	0	20.4	2.5	1.46
6	0	0	20.1	2.6	1.31
7	0	0	20.5	2.8	1.79

^a^In relation to the extract

**Fig. 2:**
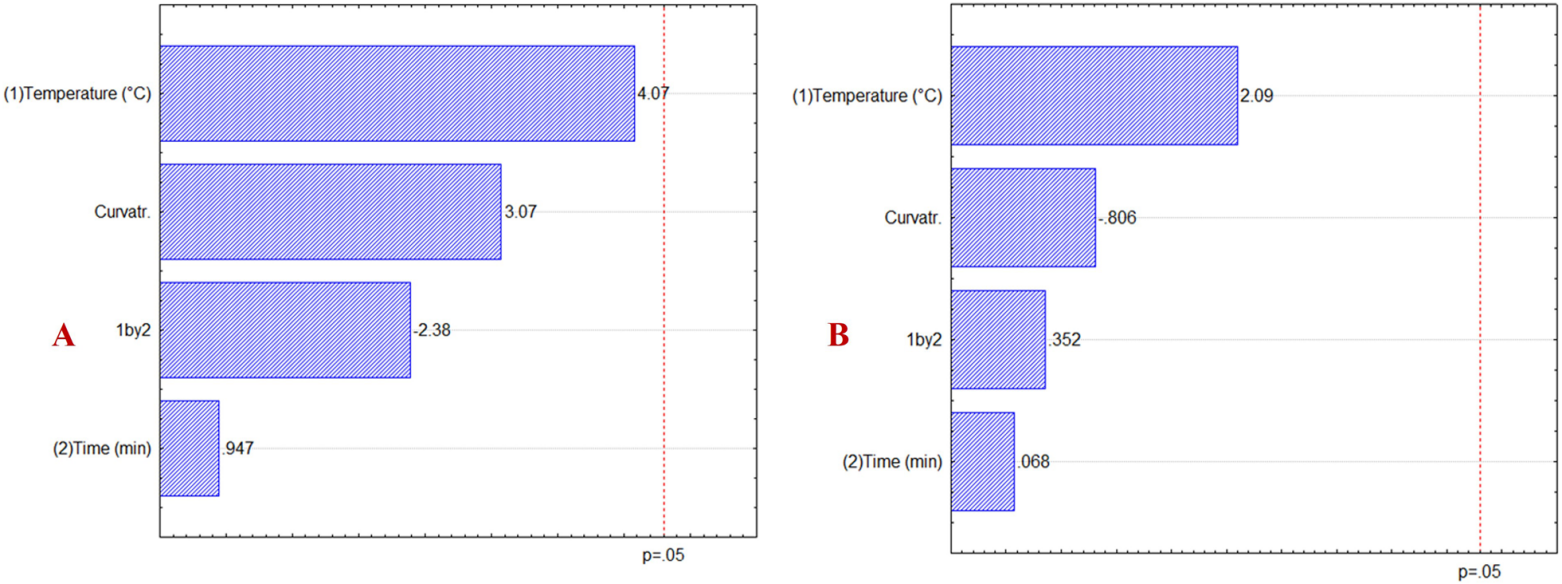
Pareto charts from the complete factorial design for results in terms of yield of bergenin from experiments using MeOH (**A**) and EtOH:H_2_O (**B**)

The Pareto graphs (Fig. 2) were obtained from the analytical responses (peak areas and yield of bergenin concerning extracts). They show that none of the factors significantly influenced the analytical response and, consequently, the bergenin extraction process. However, temperature and time had a positive effect, implying that the response increased as both factors increased. Surprisingly, when pure EtOH was employed as solvent extraction, the response was poorest than MeOH and hydroethanolic solution and the bergenin was not detected in the extracts.

None of the studied factors proved to be significant, so the values used in the central points of the mixture (115 °C and 10 min) were used as the optimal values for the next extractions.

Comparing the yields of bergenin extraction using MAE and the conventional method in terms of amounts of root was higher for MAE than for the conventional chromatographic method. The latter yielded 8.39 × 10^−2^% of bergenin, while MAE yielded 0.45% in MeOH (using the central points and the average of the root quantities, extracts, and yield of bergenin determined by HPLC). Moreover, MAE had a shorter extraction time and minimal solvent amount use.

**Fig. 3:**
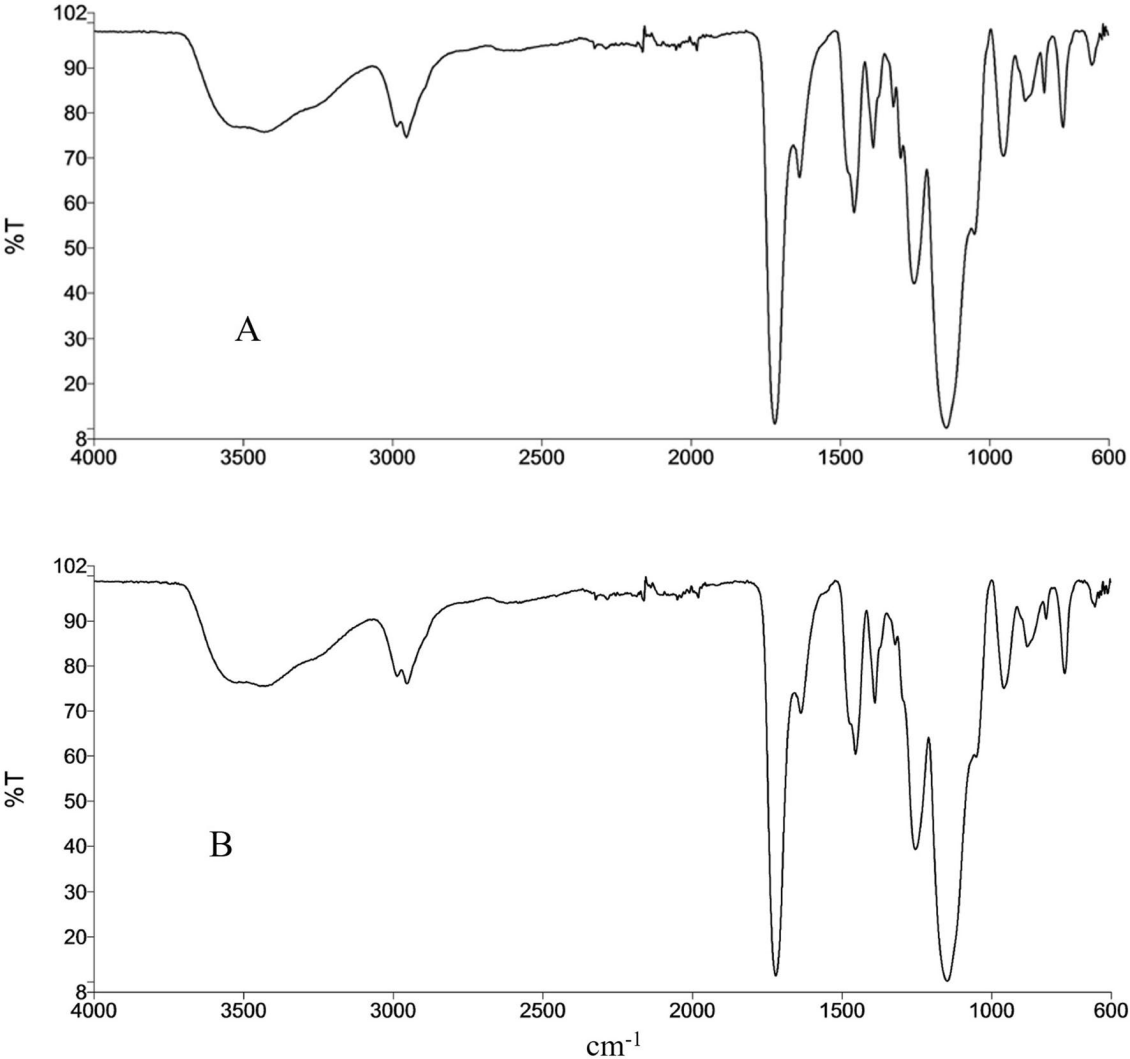
FTIR of NIP (**A**) and MIP (**B**) in ATR mode

To develop a method for isolation and purification of bergenin from crude extract, MIP based on methacrylic acid and ethylene glycol dimethacrylate using bergenin as molding compound was prepared and the extraction evaluated. The prepared MIP and NIP were characterized by Attenuated Total Reflection Fourier Transform Infrared (ATR-FTIR, Fig. 3), whose display similar characteristics. The main IR bands that characterize the printed (MIP) and non-printed (NIP) polymers can be observed, indicating that the process of addition of the template molecule did not affect the main structure of the polymer. They show the stretching bands of OH, C=O and COO groups of the carboxylic acids/esters (υ 3.430, 1720, and 1.253 cm^−1^), H–C_sp3_ methylene and methyl groups (υ 2980–2880 cm^−1^), C=C vinyl group (υ 1.637 cm^−1^), all indicative of the polymerization.

**Fig. 4:**
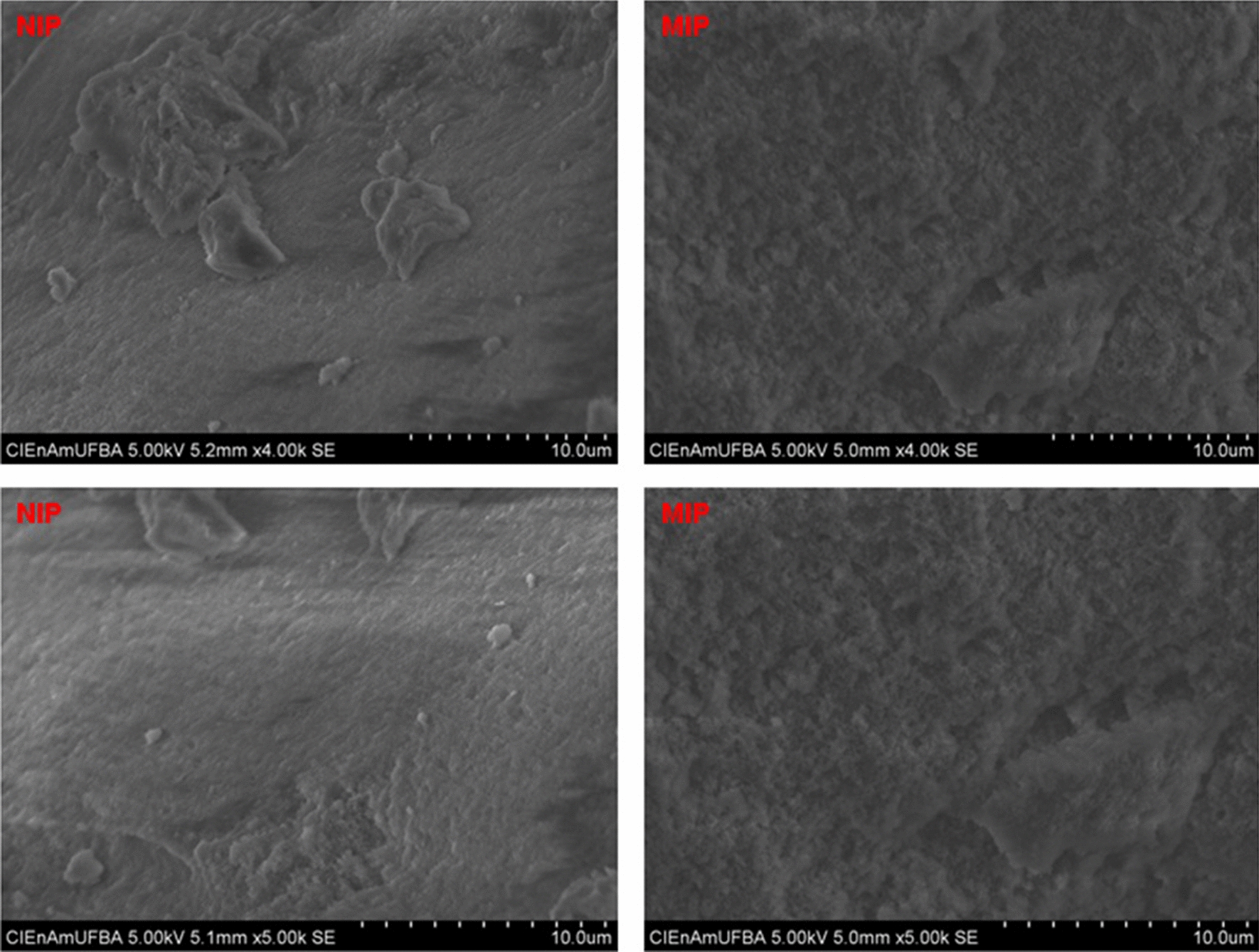
Scanning electron microscopy (SEM) images of NIP and MIP at 4000 and ×5000 amplifications

The polymer images obtained by Scanning Electron Microscopy (SEM) show the influence of bergenin as a template molecule on the morphology of polymers. Figure 4 compares the surfaces of MIP and NIP, revealing that NIP has a more compacted and smoother appearance than MIP [22]. Unlike the NIP polymer, SEM reveals that MIP has a surface with greater roughness formed by granular and porous morphology. This feature is because imprinted polymers have larger surface areas than non-imprinted ones. This structural difference suggests there will have more binding sites and which are better distributed throughout the MIP surface [23] However, the presence of irregular particles, although not considered a problem when the polymer is used in Solid Phase Extraction (SPE), makes its use as solid support in chromatographic columns not viable because the irregular particles do not pack well and create voids in the column [24].

**Fig. 5:**
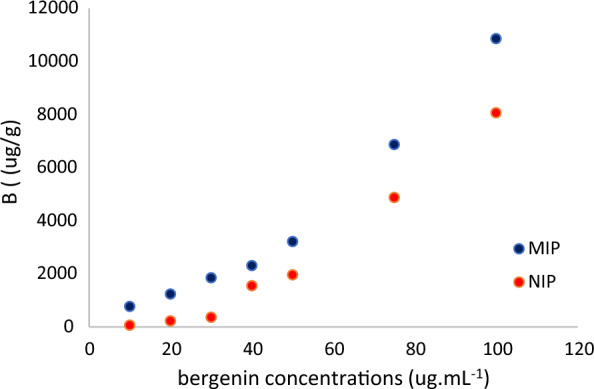
Isotherms of adsorption of bergenin by MIP and NIP

Bergenin adsorption experiments comparing MIP and NIP followed by molecular imprinted solid phase extraction (MISPE) were carried out by HPLC analysis compared with the pure standard. The variation of the content of the analyte in different prepared MeOH solutions permitted to verify the adsorption of bergenin in both polymers. The amount of bergenin adsorbed by NIP and MIP polymers was estimated (“Experimental” section) and is expressed in Table 4. The adsorption of bergenin in imprinted and non-printed polymers exhibited differences, with the analyte demonstrating a clear preference for MIP, as evidenced by the higher B^1^ value in all analyzed intervals. This characteristic can be observed in the adsorption isotherm obtained by HPLC (Fig. 5). The isotherms exhibited an increasing linear tendency, without a saturation region, where specific and non-specific binding sites are occupied, and the concentration of bergenin bound to the polymer remains constant [25].

**Table 4:** HPLC quantification of bergenin adsorption by mass of NIP and MIP

Analyte concentration (µg mL^−1^)	NIP (µg g^−1^)	MIP (µg g^−1^)
10	66.87 ± 1.42	771.59 ± 18.30
20	227.95 ± 9.79	1229.76 ± 30.85
30	366.35 ± 11.38	1828.91 ± 17.86
40	1552.59 ± 19.07	2306.47 ± 14.13
50	1963.45 ± 13.31	3216.67 ± 17.74
75	4877.35 ± 8.54	6870.61 ± 16.09
100	8069.74 ± 10.51	10859.22 ± 25.17

**Fig. 6:**
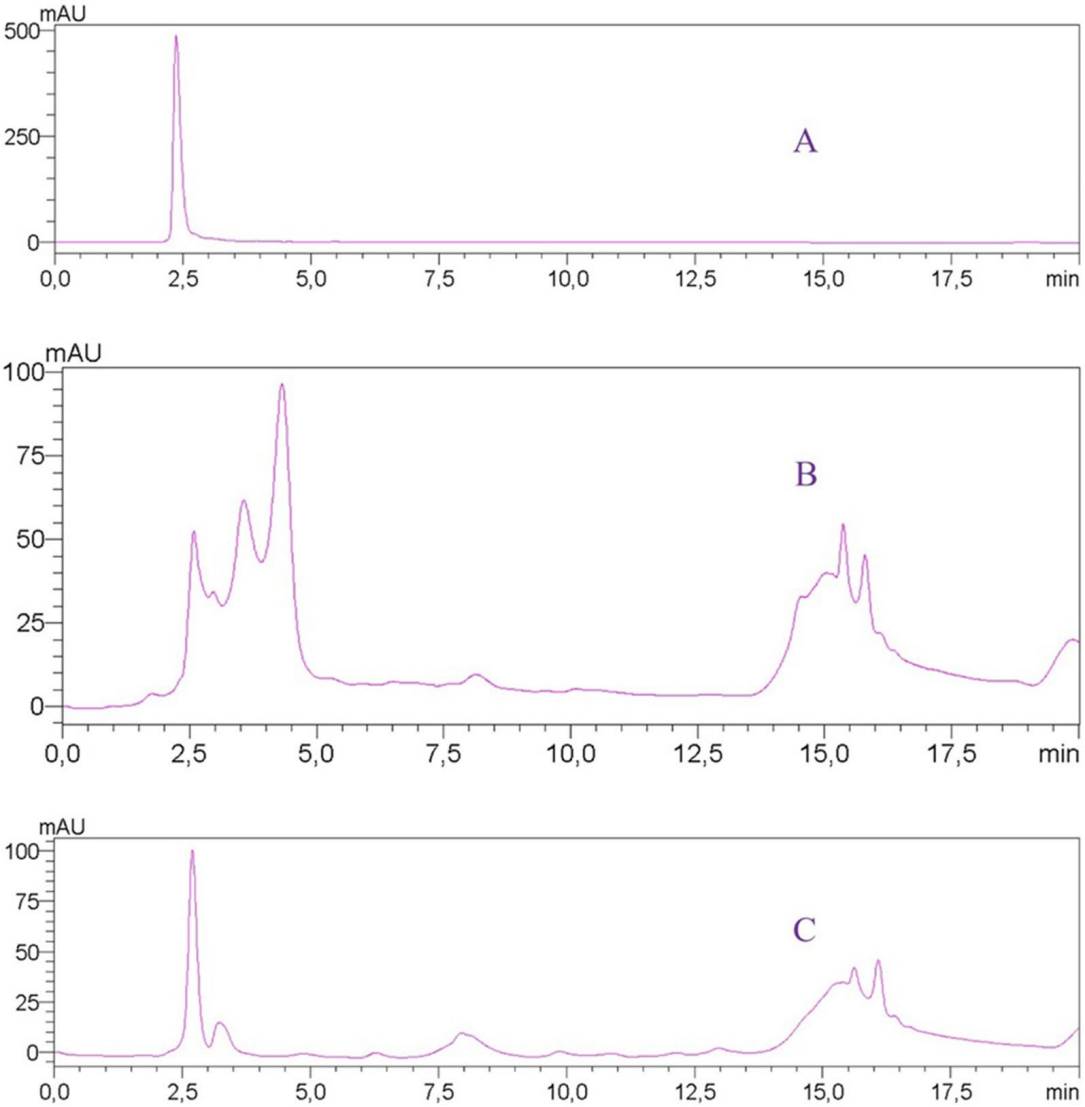
HPLC chromatogram of pure standard (**A**), the NISPE (**B**) and MISPE (**C**) eluates containing enriched bergenin (**1**)

The separation of bergenin from the extract solution (1 mg mL^−1^) by the MIP and NIP cartridges permitted to evaluate the MIP’s selectivity. Compared with the standard chromatogram, the HPLC quantification of the eluate of the two polymers in triplicate allowed the MIP to show higher selectivity for bergenin than the NIP. Besides, the chromatogram indicating some impurities in the eluate from MIP, it presented fewer interferences, thus facilitating the bergenin purification (Fig. 6).

Concerning to the dendrochronological study and based on the validated methodology, both gallic acid and bergenin were detected and quantified (Table 5) in five growth rings of a heartwood (TPD1–TPD5) of an approximately 31 years old tree, besides the phelloderm (TPD6) and barks (TPD7).

**Table 5:** Quantification of bergenin and gallic acid from *P. dubium* growth rings, barks, and phelloderm

Annual ring	Sample	Mass (g)	Extract (g)	Gallic acid (%)	Bergenin (%)
0–3rd	TPD1	1.5845	0.0964	nd	0.127 ± 0.004
5–7th	TPD2	1.5855	0.1041	nd	0.191 ± 0.007
11–14th	TPD3	1.5574	0.1486	0.029 ± 0.001	0.198 ± 0.004
17–20th	TPD4	1.5851	0.1990	nd	0.126 ± 0.006
26–28th	TPD5	1.5862	0.0917	nd	0.071 ± 0.002
Phelloderm	TPD6	1.5485	0.0721	nd	0.092 ± 0.004
Barks	TPD7	1.5508	0.1024	nd	0.026 ± 0.001

The results indicate bergenin was present in higher concentrations in the heartwood of the 11–14th growth year, and its presence in the tree diminished from heartwood to barks. Besides, different from roots, gallic acid, the biosynthetic precursor of bergenin, is not present in detectable quantities in almost all growth periods. The observed variation on specific metabolite could contribute to understanding how trees respond to environmental factors such as climate, air pollution, nutrient availability, and water stress. To date there are few studies of chemical variation in dendrochronology ring growth analysis, and it can correlate growth ring patterns with changes in the chemical composition of trees and investigate how these factors affect their development over time. For instance, the higher content of copaiba oil from *Copaiba multijuga* is found in species older than 50 years and is related to the diameter at breast height (DBH), another common technique to measure tree growth [26].

## Conclusions

The validated method proved reliable, accurate, and suitable for quantifying bergenin in MeOH extracts of *P. dubium*. It also confirmed that the adsorption of the target compound differed between imprinted and non-imprinted polymers, with the analyte showing a clear preference for MIP. However, further tests are needed to compare different monomers, adsorption amounts, and solvents. MAE using MeOH yielded higher amounts of bergenin, and temperature and time had a positive effect, meaning that the response increased with both factors. Lastly, this study suggested that bergenin was more concentrated in the heartwood of the 11–14th growth year, and its presence decreased from heartwood to barks.

## Experimental

### Instruments and software

The NMR spectra were recorded on a Bruker Avance III 500 (11.5 T) instrument at LabRMN (Universidade Federal de Goiás). A Shimadzu SPD-M20A HPLC/DAD system was used for the chromatographic analysis. The FT-IR spectra were obtained on a Perkin Frontier instrument in ATR mode. The SEM images were acquired on a Hitachi S-3400 N instrument operating at 5.0 kV (Centro Interdisciplinar de Energia e Ambiente-CIENAM/UFBA). The MAEs procedures were performed using a CEM Discover®-SP, W/Activent (SN: DC6562) instrument at a frequency range of 50–60 Hz, using the 10 mL Pyrex pressure vial for closed vessel reactions, under the indicated power automatically to reach and maintain the set temperature, specified in each case, with IR temperature control and medium stirring speed using cylindrical stir bars (10 × 3 mm), default ramp time of 10 min.

### Plant samples

*Peltophorum dubium* roots and heartwood were collected at the Ondina Campus of Universidade Federal da Bahia in Salvador, Bahia (13° 0′ 22.584″ S 38° 30′ 35.918″ W). The identification of the species were provided by Prof. Maria L. S. Guedes and a voucher is deposited in the Herbarium “Alexandre Leal Costa” of the Institute of Biology under the number #122228 (SISGEN register # AA133B8).

### Materials

The analyses by thin layer chromatography (TLC) were carried out using silica gel (SiO2) plates supported on aluminum foil (silica gel 60 F254 sheet, 0.2 mm thick, 2.5 × 7.5 cm, Riedel-deHäen® or Whatmann). The TLC plates were exposed to UV radiation in a Spectroline Model CM-10 cabinet (lamps of 254 and 365 nm). In column chromatography (CC), Acros® silica gel 60 (63–200 or 40–63 μm) were used as the stationary phase. Solvents were concentrated on IKA® RV10 Digital (40–50 °C, 100–120 rpm) and Buchi Rotavapor RII (50 °C, minimum pressure 25 mbar) rotary evaporators. The solvents (MeOH, CHCl_3_, CH_2_Cl_2_, Hex, EtOH, CAN, DMSO and EtOAc) and reagents (methacrylic acid, ethylene glycol dimethacrylate 98%, and AIBN) used in all procedures were analytical or HPLC grade (Baker, Vetec, Synth or QHEMIS). Methanol-d4 deuterated from Isotech was employed as NMR solvent The plant material was pulverized in a cutting Wiley Mill-Model 4.

### Isolation and purification of bergenin from the roots

The roots (1234.71 g) were dried in a forced circulating oven (40 °C) for 72 h, powdered in a mill and submitted to maceration in 4.0 L of MeOH (48 h) twice. After vacuum evaporation of the solvent, the MeOH extract (28.63 g) was partitioned sequentially between MeOH:H_2_O (8:2) and hexane for deffated and sequentially by CHCl_3_ (8.23 g), and EtOAc (7.32 g). The CHCl_3_ soluble fraction submitted to a chromatographic column (CC) containing silica gel 60 and it was eluted with CH_2_Cl_2_:MeOH (8:2). The fifth fraction (50 mL) furnished pure bergenin (1.037 g, 3.62% of yield) as standard.

### Multivariate optimization of bergenin extraction assisted by microwave (MAE)

All assays were performed with 0.020 g of plant material from *P. dubium*. In MAEs were employed MeOH, EtOH:H_2_O (6:4) and pure H_2_O and they were carried out under standardized conditions, with an equipment constant power of 200 W and a fixed solvent volume of 3 mL. In order to perform the multivariate optimization, temperature and time of extraction were the variable parameters selected. Table 6 details the factors with the levels low (−), mean (0) and high (+). The acquired response was the area of the peak of bergenin in the chromatogram, obtained by injecting the samples in the HPLC and the data were submitted to a statistical examination using the software Statistica 7.0.

**Table 6:** Factors and levels low, mean, and high in the MAE of bergenin

Factors	−	0	+
T (°C)	80	115	150
T (min)	5	10	15

### HPLC analysis

In the HPLC/DAD analysis of the eluates from MIP and NIP experiments was employed a XBridge BEH RPC18 (100 mmL × 3 mmI.D., 2.5 μm) column (Waters), MeOH:H_2_O (7:3) as mobile phase, and flow rate of 0.5 mL/min. The analysis were carried out in in isocratic mode from 0 a 10 min and from 10 to 13 min as gradient trough MeOH pure, totaling 20 min. The oven temperature was set of 40 ± 1 °C, and volume of injection of 5 µL and the DAD detector was set at λ 254 nm. For the dendrochronological and MAE HPLC analysis were employed a Shimadzu equipment mod. SPD-M20A and a VP-C8 Shim-pack (150 mmL × 2 mmI.D., 5 μm) column (Shimadzu). A H_2_O:MeOH (85:15) mixture was employed as eluent in a 0.25 mL/min rate (0–8 min) and gradient through MeOH from 8 to 15 min, in a total run of 20 min using a oven temperature of 40 °C. The identification of gallic acid and bergenin were identified in the extracts by comparing the retention times and UV spectra with the pure standards.

### Validation parameters

The analytical method was validated for each pattern according to the parameters of selectivity, linearity, precision, accuracy, limit of detection and limit of quantification according to procedures previously published [27]. Selectivity was determined by comparing the peaks of standard and samples analyzed, considering retention time and UV spectra observed by DAD of at least three different points of the chromatograms (beginning, half, and end peaks). Linearity was obtained by calibration curves using a correlation coefficient (R^2^). Calibration curves were obtained by triplicate injections (*n* = 3) of solutions containing six different concentrations of the external standard (5, 10, 20, 30, 40 and 50 µg/mL). Peak areas were correlated with the averages of each concentration, and a graph was plotted using the least squares method. Precision was determined by injection in triplicate of three solutions of the standards. This parameter was expressed as the relative standard deviation according to the equation RS(%) = SD/AC ∗ 100, where SD is the standard deviation and AC is the average concentration determined. The recovery factor verified the accuracy, where samples with no analytes were spiked with standard solutions of low, medium, and high concentrations. The spiked samples were subjected to the whole extraction process and injected into HPLC. The following equation determined the accuracy: Rec(%) = [obtained concentration]/[absolute concentration] ∗ 100. The detection limit (LoD) and quantification limit (LoQ) were estimated by the ratio of standard deviations and slopes of calibration curves, according to the equations LoD = SDa ∗ 3/S and LoQ = SDa ∗ 10/S, where SDa is the standard deviation obtained from the calibration curve and S is the curve’s slope. Finally, the robustness assessment performed deliberate changes only in the mobile phase flow rate and temperature. Thus, it is noteworthy that this evaluation was simplified without involving a more detailed statistical treatment so that the chromatograms obtained from the corresponding Rt values and UV spectra were compared.

### Synthesis of the MIP and NIP

The MIPs’ synthesis was realized as bulk adapted from previously reported procedure [28]. The MIP mold molecule (bergenin) was solubilized (0.8 mmol, 0.263 g), in 10 mL of DMSO/acetonitrile (1:1). In sequence, methacrylic acid (4 mmol, 0.344 g) was added in the obtained solution, ethylene glycol dimethacrylate (20 mmol, 3.96 g), the cross-linked reagent, and AIBN (0.131 g) as radical initiator. The reaction was kept under heating at 60 °C, in an inert nitrogen atmosphere, and stirring for 24 h.

### Extraction of bergenin by MIP/SPE and analysis of the adsorption

Empty polypropylene SPE cartridges (6 cm × 1 cm) were filled with 100 mg of MIP or NIP between two frits at the top and the bottom of the polymer layer. These MIP cartridges were conditioned with 3 mL of MeOH followed by deionized H_2_O employing a Manifold to elute the solvents. In sequence, a solution of 0.5 mg/mL of aqueous MeOH of *P. dubium* root MeOH extract was eluted in the cartridge. The analyte was eluted with 2 mL of MeOH, and the content of bergenin was analyzed by HPLC/DAD.

In order to develop the adsorption isotherms, 10 mg samples of MIP and NIP were subjected to agitation for 1 h in a container equipped with a magnetic stirrer, containing 2 mL of a bergenin solution in different concentrations (10, 20, 30, 40, 50, 75 and 100 µg/mL). All solutions were prepared using methanol as solvent. Thus, the amount of bergenin adsorbed by the polymers MIP and NIP was estimated using the following equation:

1 $$\frac{B=\left(I-F\right) \cdot V}{{m}_{polymer}}$$

where *B* is the adsorbed bergenin, *I* is the initial concentration of the solution (µg/mL); *F* is the concentration of bergenin in solution (µg/mL) after the adsorption procedure; *V* is the volume of solution containing bergenin used (mL); and *m*_polym_ is mass of the MIP/NIP (g).

### Dendrochronological analysis of the tree and sampling of the heartwood

**Fig. 7:**
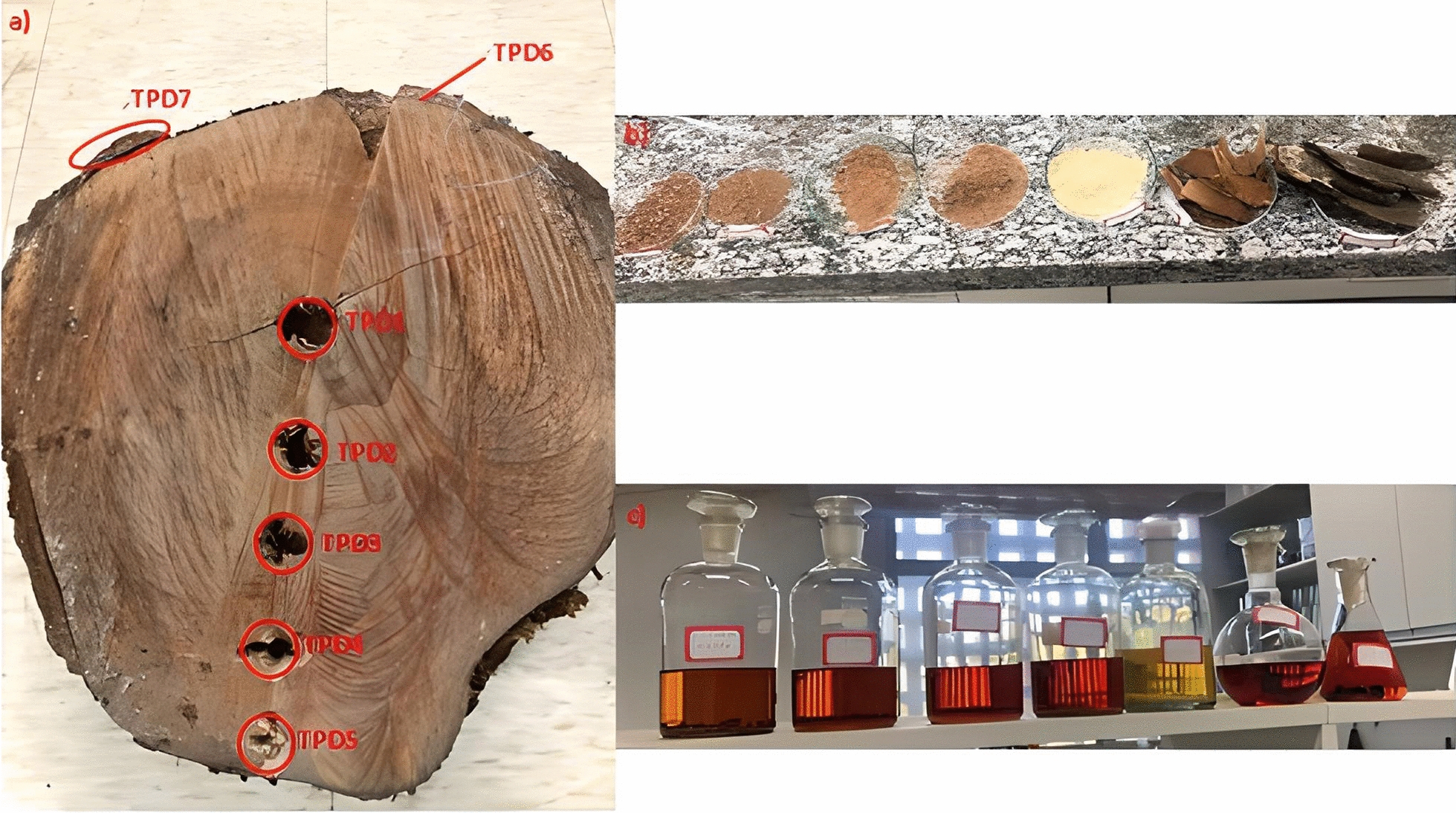
Sample of trunk heartwood of *P. dubim* and the seven samples obtained from medulla to bark (TPD1–TPD7)

Sample of the trunk of *P. dubium* was collected at a height of 20 cm from the base, with a diameter of 32.4 cm and a circumference of 100.5 cm. The trunk section was sanded to improve the visibility of the growth rings. The dendrochronological analysis was followed by sampling at seven points, with the first five points ranging from the nucleus (center, indicating the year of germination) to the phloem region, at an interval of 4.5 ± 0.1 cm, and the last two points in the phelloderm and bark (Fig. 7). The samples were macerated with MeOH for three days and dried under reduced pressure. The bergenin content was quantified by HPLC, according to the method previously described.

### Supplementary Information

**Additional file 1.** The bergenin spectra, NMR data and other informations can be obatained.

## Data Availability

All data generated, discussed, or analyzed during the development of the present study are included in this current article or in Additional files.

## Footnotes

1 Adsorved bergenin.
