# Supplementary material for: Microwave extraction and molecular imprinted polymer isolation of bergenin applied to the dendrochronological chemical study of Peltophorum dubium
Source: BMC Chem. 2024 Jan 13;18(1):13. doi: 10.1186/s13065-024-01112-7 (PMC10788031; doi:10.1186/s13065-024-01112-7)
Supplement: Supplementary file 1 — Additional file 1. The bergenin spectra, NMR data and other informations can be obatained. [file 13065_2024_1112_MOESM1_ESM.docx]

**Microwave extraction and Molecular Imprinted Polymer Isolation of Bergenin applied to Dendrochronological Chemical Study of *Peltophorum dubium***

Oscar Caetano Silva Neto, Caio Silva Assis Felix, Leonardo de Oliveira Aguiar, Maurício Brandão dos Santos, Silvio Cunha, Jorge Mauricio David^*^

Instituto de Química, Universidade Federal da Bahia, campus Ondina, 40170280, Salvador (BA) Brazil.

*correspondence author ([jmdavid@ufba.br](mailto:jmdavid@ufba.br))


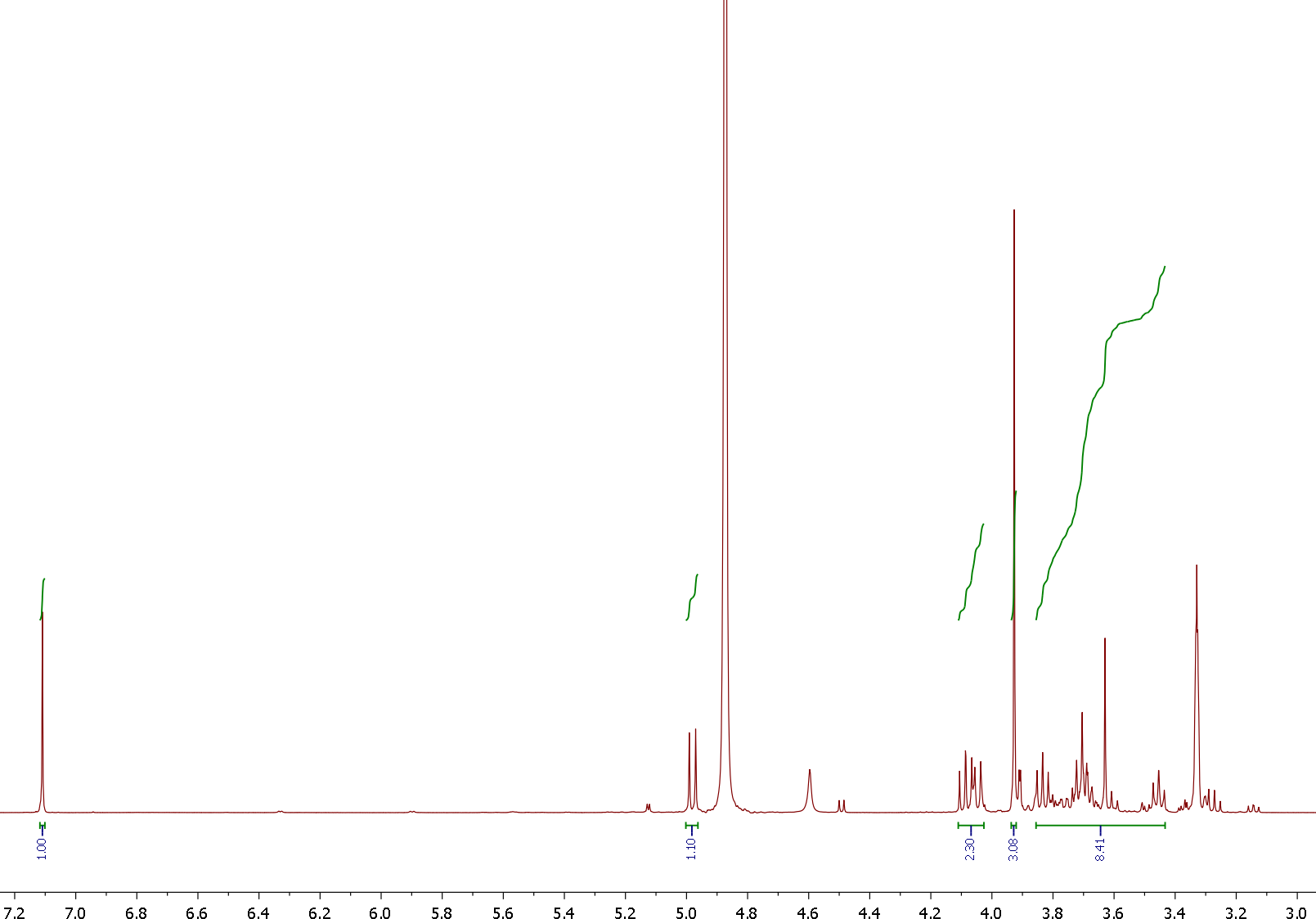


**Figure 1S** ^1^H NMR spectrum of bergenin (**1**) [500 MHz, CD3OD, δ (ppm)].


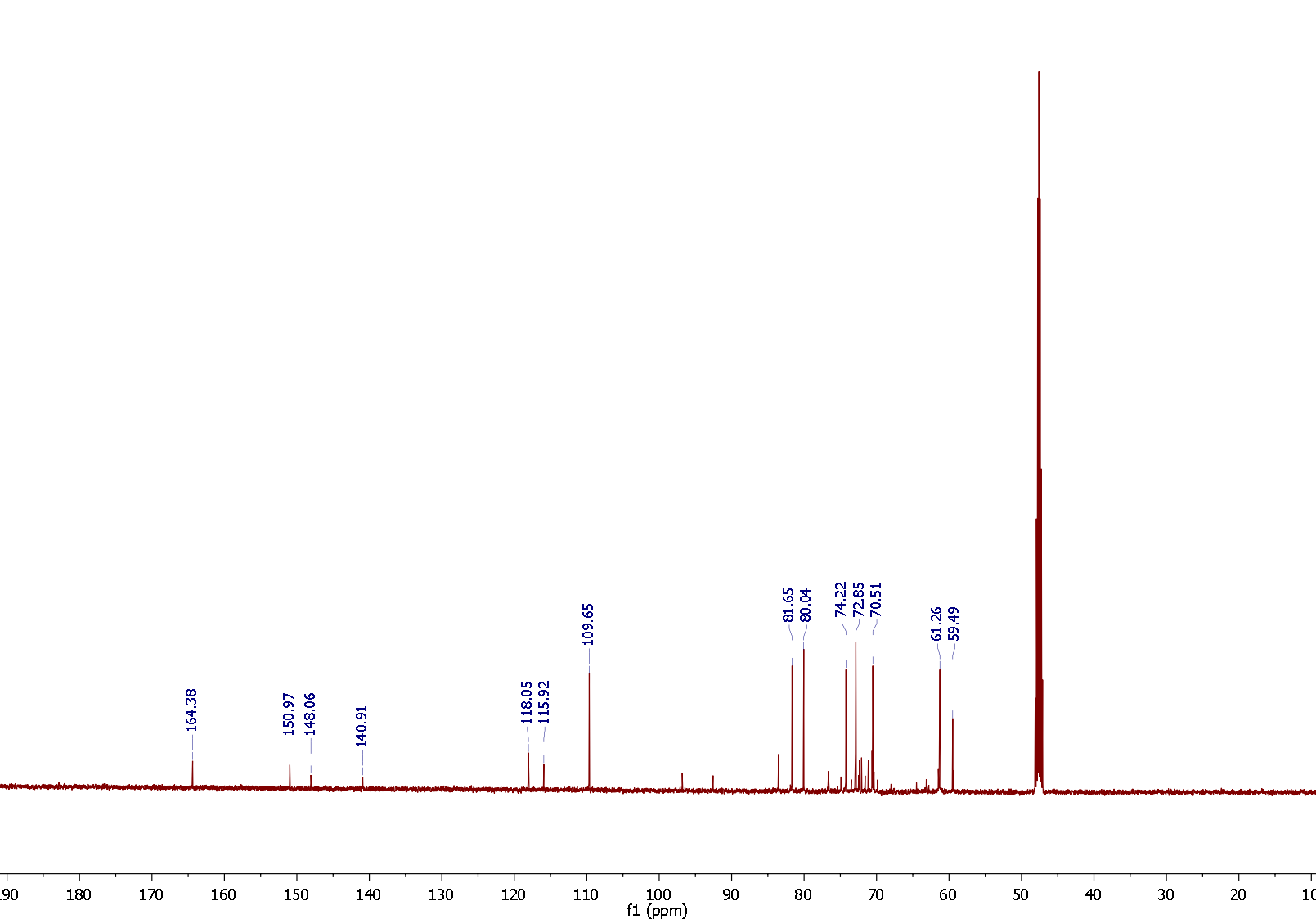


**Figure 2S:** ^13^C NMR spectrum of bergenin (**1**) [125 MHz, CD_3_OD, δ (ppm)].


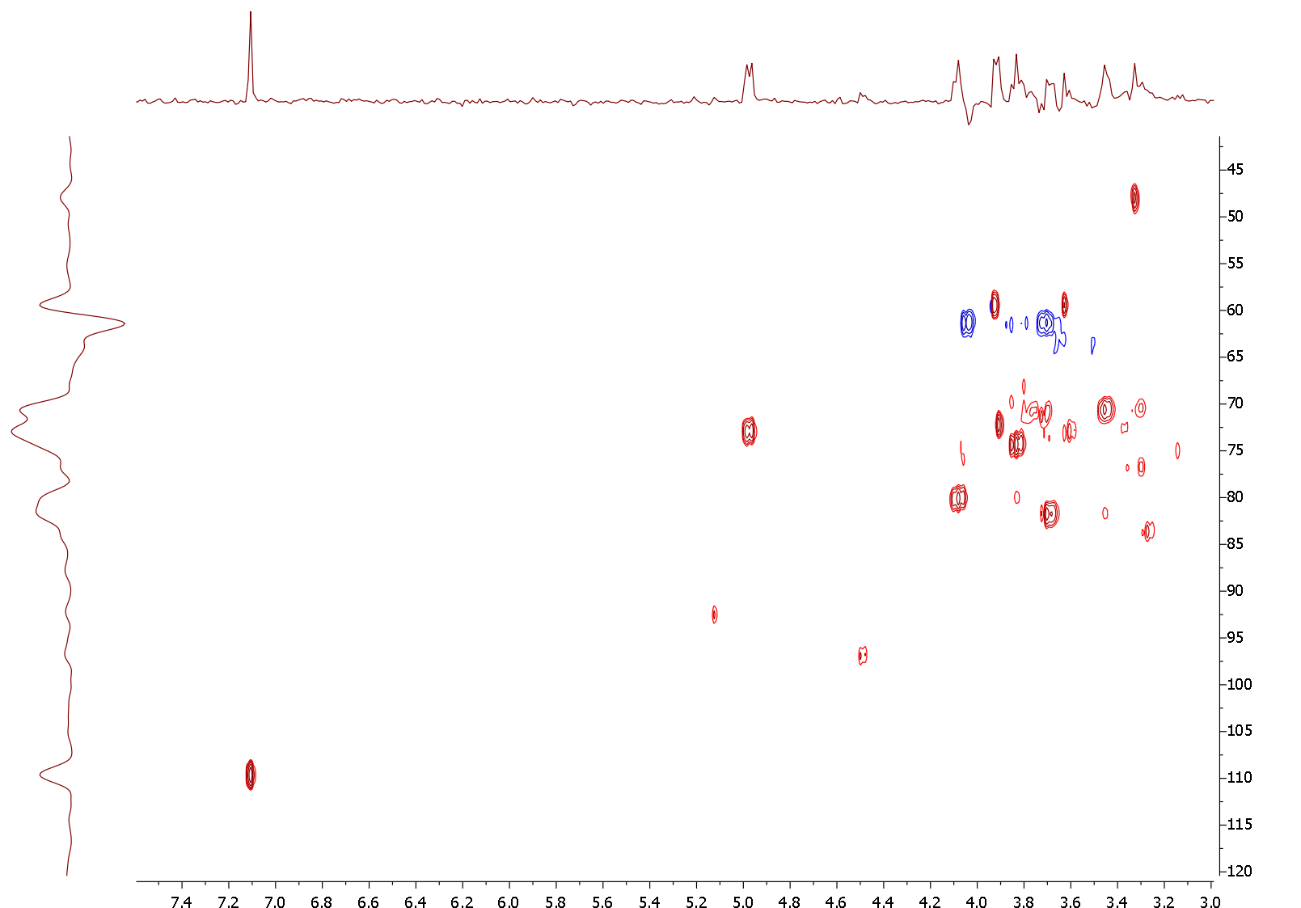


**Figure 3S**: HSQC contour map of bergenin (**1**) [500/125 MHz, CD_3_OD, δ (ppm)].


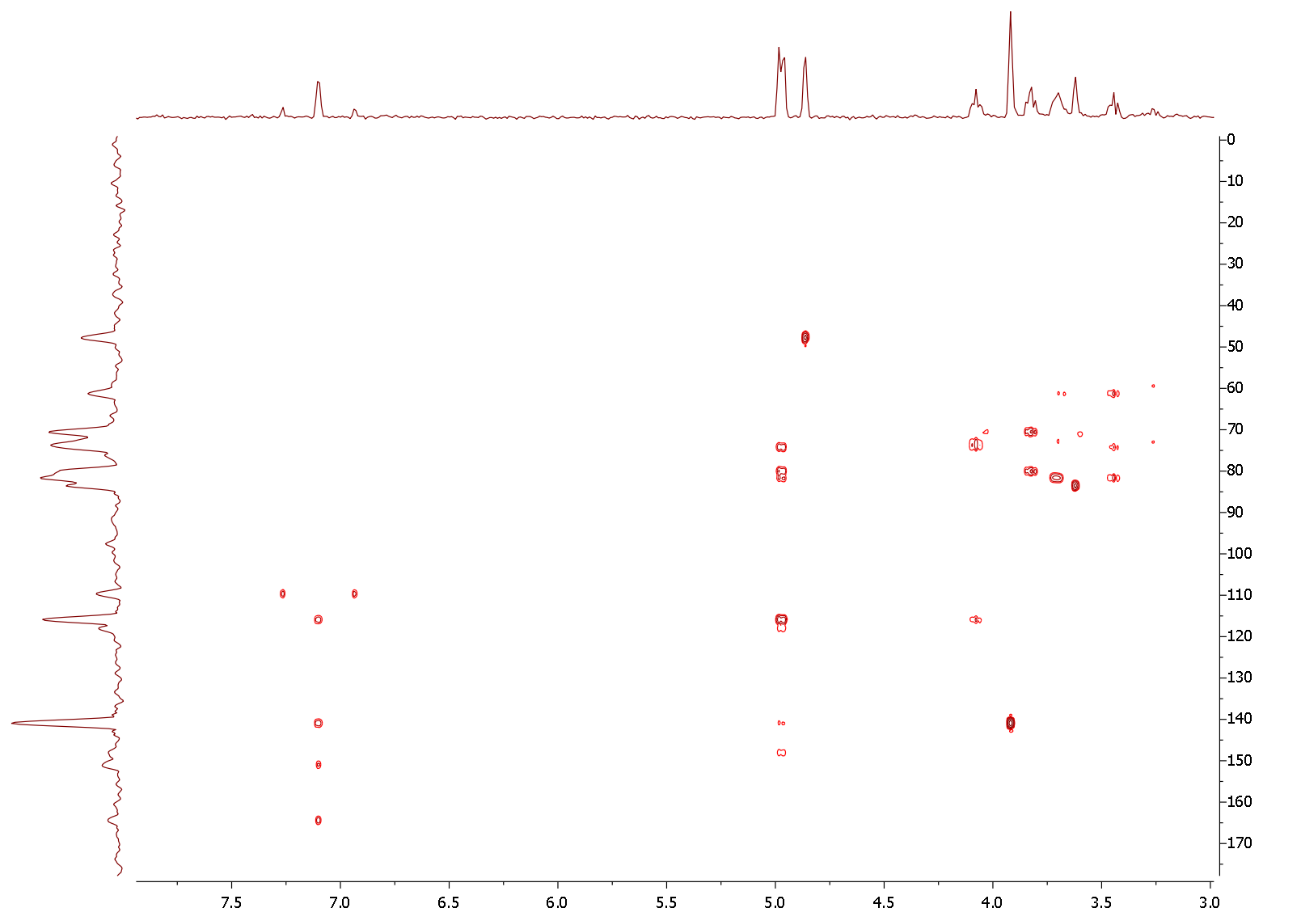


**Figure 4S**: HMBC contour map of bergenin (**1**) [500/125 MHz, CD_3_OD, δ (ppm)].

**Table 1S:** ^1^H (500 MHz) and ^13^C (125 MHz) NMR data of bergenin [CD_3_OD, δ (ppm), J (Hz)].

|  | ^1^H | ^13^C |
| --- | --- | --- |
| 2 | 3.44 – 4.11 (m) | 81.65 |
| 3 | 3.44 – 4.11 (m) | 70.51 |
| 4 | 3.44 – 4.11 (m) | 80.04 |
| 4b | 3.44 – 4.11 (m) | 74.22 |
| 10b | 4.98 (d; 10.3) | 72.85 |
| 10a | - | 115.92 |
| 6 | - | 164.38 |
| 6a | - | 118.05 |
| 7 | 7.11 (s) | 109.65 |
| 8 | - | 148.06 |
| 9 | - | 140.91 |
| 10 | - | 150.97 |
| 11 | 3.44 – 4.11 (m) | 61.26 |
| MeO | 3.93 (s) | 59.49 |
